# The future of routine immunization in Africa

**DOI:** 10.11604/pamj.supp.2017.27.3.13054

**Published:** 2017-06-21

**Authors:** Raoul Kamadjeu

**Affiliations:** 1 UNICEF Eastern and Southern Africa Regional Office (ESARO), Nairobi, Kenya

**Keywords:** Expanded program on immunization, vaccine preventable diseases, immunization

## Commentary

The Addis Declaration [1] adopted at the first Ministerial Conference on Immunization in Africa re-iterates the critical value of vaccines in improving the health of people on the continent. In Africa, the successful implementation of vaccination programs resulted in a drastic reduction of the burden of vaccine preventable diseases (VPD): polio is on the verge of being eradicated, more than thousands of measles cases and deaths have been averted; neonatal tetanus is close to elimination and the dreaded cycle of Meningitis A epidemics in West Africa has been broken; to name just a few. Most vaccinations in Africa are delivered through the Expanded Program on Immunization (EPI); a combination of infrastructural, programmatic and financial processes aiming at systematically delivering the much needed vaccines to all eligible target population in a country.

EPI is a legacy of the successful campaign that led to the eradication of smallpox [2]. In 1974, the World Health Organization recommended the inclusion of additional vaccines to be delivered through the smallpox eradication platform [3]. The first diseases targeted by EPI were diphtheria, measles, poliomyelitis, tetanus, tuberculosis and whooping cough. By the mid-eighties, most countries in Africa were routinely providing immunization against the six traditional diseases; the expanded program on immunization thereof assumed the shape it has today. The first decade of EPI brought the finalization of the standardized EPI schedule, a gradual adoption of the program by WHO member states and the establishment of the goal of providing universal immunization for all children by 1990.

EPI then went through a period of maturation that involved various levels of sophistication aimed at improving coverage, reaching previously unreached children, ensuring equitable access to vaccine and introducing new or underused vaccines. Some events have accelerated the transformation of EPI more than others; the creation of the Global Alliance for Vaccine and Immunization (Gavi) in 2000 [4] marked undoubtedly a drastic turn in the coming of EPI into adulthood. Gavi is a global vaccine alliance bringing together governments, private sectors, UN agencies, implementing countries and other bodies that shared the goal of creating equal access to new and underused vaccine for children in the world’s poorest countries. Through its business model, its ability to shape the global market for vaccines and its overall support to health and immunization systems, Gavi has played and continues to play a major role in expanding the reach and offer of EPI vaccines. Gavi estimates that it supported over 200 vaccine introductions and campaigns between 2011 and 2015 [4]. With the introduction of Pentavalent (Penta), Meningitis A, Rotavirus, Human papillomavirus, Measles-Rubella, Pneumococcal Conjugated and Inactivated Polio vaccines to name most important, the typical EPI schedule in Africa now includes close to 12 antigens.

Albeit large cross-national variations in national immunization coverage across the continent, EPI in it forth decade boasts commendable achievements in VPD control and elimination. The variable performance of the EPI program across Africa reflects most often a variety of countries implementation frameworks where factors such as insecurity/inaccessibility, structural health systems performance and overall governance environment play a key role.

Reaching more people, with more vaccine, in an equitable way remains by far the biggest challenge of the EPI program in Africa as it enters adulthood: since the early eighties when EPI data were systematically compiled in the WHO/UNICEF Estimates for National Immunization Coverages (WUENIC) [5], the average coverage for the third dose of DTP/Pentavalent vaccine for 47 African member states has not exceeded 80%. In 2015, only 27 countries (57%) out of the 47 had an estimated Penta-3 (DTP 3) coverage above 80%. Even more worryingly, the number of countries with Penta-3 above 80% has barely budged over the last 10 years (from 21 in 2005 to 27 in 2015). On a different end of the coverage spectrum, high coverage at national level sometimes blurs a tapestry of acute sub-national disparities in coverage and inequitable access to vaccine by groups or communities.

The performance of the EPI program cannot be decoupled from the performance of the health system in which it emanates (operating environment); poor EPI performance often translates structural weaknesses in health systems. Sustained and predictable financing, peace and security, strategic partnerships and overall governance mind-set are important determinants of this operating environment. Health policy frameworks such as Agenda 2063: the Africa We Want [6], The Africa Health Strategy 2015 – 2030 [7] and other global commitment like the Sustainable Development Goals [8] have the potential to nurture an enabling environment for health programs in Africa, including EPI. EPI will equally be infused by potentially transformative forces such as:

- The advent of new vaccine technologies (needle free vaccine administration, cold chain independent vaccines) have the potential to expand the reach of the program by freeing it from stringent cold chain and technical know-how requirements.
- The introduction of more new or underused vaccines: will extend the opportunity to protect more people against more diseases throughout the course of life
- The adoption of information technology around supply chain management, population based registries, personalized vaccination records, reminder systems, etc… will push the EPI program into the digital age.

EPI will be humbled by the imperative to find innovative ways to sustain community awareness on the benefits of vaccination, to increase demand and offer of services, to strengthen confidence in the program and to better communicate risk.

The future health landscape of Africa in full of challenges: the prevalence of non-communicable diseases will continue to rise; the burden of cancer is expected to double between 2008 and 2020; injury and trauma will increasingly contribute to disease burden; challenges link to climate change, food insecurity, conflicts and water scarcity will increase. With this in mind, vaccines provided through EPI are the weapons at hands to remove VPD from the pool of health challenges the continent will grapple with in the future; this opportunity cannot be wasted.

## Competing interests

The author declares no competing interest.
